# Editorial: Environmental health in informal settlements: current progress, challenges and perspectives

**DOI:** 10.3389/fpubh.2023.1200422

**Published:** 2023-08-02

**Authors:** Yutong Samuel Cai, Adetoun Mustapha

**Affiliations:** ^1^Centre for Environmental Health and Sustainability, University of Leicester, Leicester, United Kingdom; ^2^Nigerian Institute of Medical Research, Lagos, Nigeria

**Keywords:** informal settlement, urban health, environmental health, SDG, environmental exposure

It is estimated that about two billion people will live in urban informal settlements by 2030, mainly in sub-Saharan Africa and southern and southeast Asia (1). Majority of these populations will reside in small-to-medium-size cities in low-and-middle-income countries (LMICs), where rapid urbanization continues without adequate provision of infrastructure for healthcare, housing, waste management, transport, and emergency services. Therefore, populations of informal settlements are exposed to elevated health risks and often face socioeconomic, environmental, and legal-political exclusions from the rest of the population. Due to these various exclusions, they may be disproportionately exposed to some environmental and occupational hazards, for example, indoor and outdoor air pollution, heavy traffic, road accidents, pesticides, soil pollution, infection, heat, flooding, and amongst many others. In fact, this neighborhood effect on the health of those living in informal settlements is driven by a combination of poverty and shared physical and social environment (2). However, current research on the health impacts of environmental hazards in informal settlements is still largely underdeveloped, representing a major gap in policy interventions to improve health of these marginalized populations. Considering this, we launched this Research Topic as a platform to collect and share latest research and policy insights into this area, with an aim to stimulate wider, in-depth discussion and future studies in different contexts. This Research Topic has received four research articles investigating broad environmental health in informal settlements.

Simiyu et al. presented a case study of fecal sludge management in low-income settlements in the booming town of Nakuru, Kenya. One of the key milestones in the Sustainable Development Goals (SDGs) is clean water and sanitation. Around the world, 4.5 billion people still lack safely managed sanitation service, representing an urgent issue to be addressed. However, the practices and challenges in relation to the sanitation service have rarely been documented in a low-income setting. Using qualitative methods via interviews and focused group discussions, this study has presented insightful results through each step of the sanitation value chain. Many challenges and potential solutions have been discussed through the chain, which are useful to inform policy-making, not only at local level, but also at national and international levels where contexts are similar.

Children living close to a farm and/or involved in farming activities are common in LMICs settings. Because of their immature metabolic and immune functions, children are susceptible to a higher risk of pesticide exposure, which might have lifelong health implications. In Ranga Reddy district of Telangana in India, Medithi et al. conducted a study among 129 children of aged 9–15 years to investigate whether micronutrient supplementation could be useful to mitigate the harmful impacts from pesticide exposure. They observed that blood pesticide residue levels were significantly lower after micronutrient supplementation, indicating that such relatively simple intervention might have helped enhancing pesticide metabolism and facilitating the elimination of residues. This is a small-scale pilot study; however, it sheds some lights into the role of nutrition in mitigating the harmful health impacts from pesticide exposure. Further large-scale interventional studies are warranted to validate these findings.

In Nigeria, infant vaccination uptake is one of the lowest in the world. Infants (i.e., <24 months) from the most poorest households in the country have a consistently lower rate of vaccination than other socioeconomic groups, and many of them are residing in urban slums. Sub-optimal rate of vaccination among this age group of young children can have serious impacts on their own health, herd immunity of the entire community and their life chances. In practice, older women have an important role in caring infants in Nigerian urban slums. Balogun et al. conducted an experimental study to compare the timeliness and completion of infant vaccination among infants who were taken care of older women, who were either educated of vaccination importance or not, in seven urban slums. They found that the timeliness and completion of vaccination in the intervention group (*n* = 109) was 67.9%, significantly higher than the non-intervention group (*n* = 93; 36.6%). This small-scale study provided some evidence that the educational intervention on older women can improve the vaccination uptake among infants in urban slums, however, large-scale study would be needed to test this intervention.

Finally, Adegun presented a policy brief illustrating various actionable recommendations on how green infrastructures can improve quality of life in informal settlements. The policy brief was based on a case study of three slum areas with different levels of green infrastructures in Johannesburg. Green infrastructure in informal settlements is associated with many benefits, including as a source of food (e.g., garden produce), as a mean of social interaction, and regulation of microclimate (e.g., temperature, air pollution, flooding, and wind). These benefits can directly or indirectly improve health of residents in informal settlements, especially those living on lower household incomes. However, this policy brief also highlighted some practical challenges when implementing green infrastructure in informal settlements, such as insecure land tenure, space limitation, soil condition, water resources etc. From public health as well as environmental justice perspectives, one key policy implication is to strengthen urban greening considerations within national policies that guide housing development, slum upgrading, and management of informal settlements.

To conclude, each of these four articles presented a case-study highlighting environmental health challenges and their possible solutions in different informal settlements across LMICs. However, current research on this Research Topic is still largely lagging, leaving a policy gap to improve environmental health in these vulnerable communities. With the projected increase in populations of urban informal settlements, and the fact that global climate change is affecting where and how people live, we call for a diverse range of environmental health research in informal settlements. This is necessary to bring these settlements into mainstream urban planning and healthcare resources mobilization for infrastructural development, and support health policies to address socioeconomic disadvantages that predispose these individuals to environmental pollution and ill-health.

## Author contributions

YC writing original draft of this editorial. All authors contributed to this Research Topic design, manuscript editing efforts, article, and approved the submitted version.
